# Improving blood glucose in late preterm and small for gestational age infants: the use of dextrose 40% gel

**DOI:** 10.3389/fped.2025.1591567

**Published:** 2025-06-24

**Authors:** Paola Polo Perucchin, Elena Aldera, Maria Grazia Calevo, Bianca De Grande, Monica Russo, Elisabetta Godano, Mohamad Maghnie, Cesare Arioni

**Affiliations:** ^1^ Neonatology Unit, IRCCS Ospedale Policlinico San Martino, Genoa, IT, Italy; ^2^ Paediatric Unit, Ospedale Cardinal Massaia, Asti, IT, Italy; ^3^ Epidemiology and Biostatistics Unit, Scientific Directorate, IRCCS Istituto Giannina Gaslini, Genoa, IT, Italy; ^4^ Department of Neuroscience, Rehabilitation, Ophthalmology, Genetics, Maternal and Child Health, University of Genova, Genoa, IT, Italy; ^5^ Department of Pediatrics, IRCCS Istituto Giannina Gaslini, Genoa, IT, Italy

**Keywords:** hypoglycemia, oral dextrose gel, late preterm, small for gestational age, large for gestational age, children of diabetic mothers

## Abstract

**Introduction:**

Limited evidence exists on whether administering oral dextrose gel immediately after birth reduces the risk of hypoglycemia in the early hours of life. The primary objective of this study was to assess whether early administration of 40% dextrose gel in infants with risk factors could reduce the incidence of hypoglycemia during the first few hours after birth. A secondary aim was to evaluate the impact of early dextrose gel administration on breastfeeding outcomes.

**Methods:**

This was a double-arm, randomized trial conducted in two phases that included a total of 297 patients. In the first phase, 200 infants at risk for hypoglycemia were recruited including those who were small for gestational age (SGA), late preterm (LP, born between 34 + 0 and 36 + 6 weeks), large for gestational age (LGA), and infants of diabetic mothers: 100 infants were assigned to the “Dextrose group” and received 40% dextrose oral gel 15 minutes after birth, 100 infants in the “Control group” did not receive any dextrose. Capillary blood glucose was measured at 2 and 4 hours of life. Based on the preliminary findings, the second phase of the study randomized an additional 97 LP infants: 50 in the dextrose group and 47 in the control group, following the same intervention protocol.

**Results:**

In the first phase, no significant differences in blood glucose levels were found at 2 and 4 hours of life in infants of diabetic mothers or those who were LGA. In SGA infants blood glucose levels tended to decrease significantly between 2 and 4 hours in the control group. In the second phase, LP infants who received dextrose had significantly higher blood glucose at 2 hours compared to those in the control group. Additionally, LP infants in the dextrose group who were breastfed within the first two hours showed significantly higher blood glucose at two hours than those in the control group.

**Discussion:**

Early administration of 40% dextrose gel may be beneficial in maintaining higher blood glucose levels during the first hours of life in LP and SGA infants; our findings suggest that only in LP and SGA patients early intervention with dextrose gel could support glucose homeostasis and potentially improve breastfeeding outcomes.

## Introduction

Glucose is the primary energy source for both the fetus and the newborn. During pregnancy, maternal glucose is transferred to the fetus through placental circulation, but after birth, this transfer is abruptly interrupted. In the first two hours of life, blood glucose levels can drop to their lowest point. This transient period of hypoglycemia reflects the time needed for the newborn to start independent energy production and is generally considered temporary (1, 2), it usually does not cause symptoms because, there is a physiological metabolic adaptation involving a shift in hormonal regulation, which actives hyperglycemic hormones (glucagon, cortisol, catecholamines) that stimulate glycogenolysis. After the first 12 hours of life, glycogen stores are depleted, and blood glucose levels are maintained by gluconeogenesis (1, 2).

Typically, by 4–6 hours after birth, glucose levels stabilize around 40–80 mg/dl (2,2–4,4 mmol/L) (2, 3). Normal blood glucose levels in newborns depend on the presence of glycogen stores, the maturity of enzymatic activities for glycogenolysis and gluconeogenesis, and the integration of endocrinological and metabolic responses. However, in some cases, metabolic adaptation may not occur at the proper timing due to factors that can disrupt glucose homeostasis, either temporarily or permanently (1, 2). Certain groups of newborns are at greater risk of developing hypoglycemia because the compensatory mechanisms for hypoglycemia fail to establish properly and promptly (1, 2, 4).

Neonates at risk for hypoglycemia include those preterm, SGA (defined with a birth weight <10° percentile based on INeS charts (5), LGA (defined as a birthweight >90th percentile on INeS charts (5), neonates born to women with diabetes, stressed newborns (such as those with infections, sepsis, heart or pulmonary diseases, hypothermia, hypoxia, or polycythemia), infants born to mothers who have taken medications with hypoglycemic effect (e.g.,beta mimetic, beta blockers, sulfonylureas, thiazide diuretics, tricyclic antidepressants, or maternal glucose infusion during labor), newborns with feeding disorders, or those with endocrinological or metabolic disorders. Additionally, neonates with hyperinsulinism are at higher risk (1, 2, 4, 6–8).

There is no universally agreed-upon threshold for defining neonatal hypoglycemia (3, 9). Newborns in good health may not exhibit symptoms even if hypoglycemia occurs because they can use alternative energy substrates, such as ketone bodies, released from adipose tissue (1, 2). However, it is critical to maintain blood glucose levels within a safe range appropriated for the newborn's age to avoid the risk of brain damage (10–13). According to the American Academy of Pediatrics (AAP) guidelines of 2011 (4), blood glucose values below 25 mg/dl in the first 4 hours of life are considered unsafe and must be treated with intravenous glucose monohydrate administration at a dose of 200 mg/kg (2 ml/kg), followed by a continuous infusion at 5–8 mg/kg/min (80–100 ml/kg/day) (4).

Asymptomatic hypoglycemia is typically managed with oral feeding based on blood glucose levels, with a preference for breastfeeding or formula supplementation when human milk is unavailable. If oral feeding fails to correct the hypoglycemia, intravenous glucose administration is the next step. In more recent guidelines (14–17) 40% oral dextrose gel has gained popularity and is increasingly recommended as a first-line treatment for asymptomatic neonatal hypoglycemia. The 40% oral Dextrose gel is a concentrated dextrose solution that is applied directly to the mucosal surfaces of the mouth (such as the cheeks and tongue). This method allows for rapid glucose absorption through the oral mucosa, bypassing the liver and reaching the systemic circulation quickly. The recommended dose for 40% oral dextrose gel (200 mg/kg/dose) is similar to the dose used for intravenous bolus of 10% glucose and is effective at raising blood glucose levels without causing hyperglycemia (18). Several studies have assessed the effectiveness of dextrose gel in treating neonatal hypoglycemia (18–23). However, it remains uncertain whether dextrose gel reduces the need for intravenous treatment compared to a placebo. The available data suggests that dextrose gel may help reduce mother-infant separation and increase the likelihood of exclusive breastfeeding at discharge (24). Recent studies have also explored the preventive use of 40% dextrose gel (25, 26).

In one study, newborns with risk factors were randomized to different dosage regimens of dextrose gel or placebo, with the results indicating a reduced incidence of hypoglycemia in the group that received dextrose gel, regardless of the dosage regimes (26). In a study of Harding et al. (25), newborns with risk factors were randomized to receive a single dose of 40% dextrose gel or a placebo, and again, a reduced incidence of hypoglycemia was observed in the group that received the gel. In both studies, the gel was massaged into the buccal mucosa one hour after birth, followed by breastfeeding. These findings were reviewed in a meta-analysis, which confirmed with high certainty that dextrose gel reduces the risk of neonatal hypoglycemia (27).

In conclusion, over the last 10–15 years, various studies have compared the use of 40% dextrose gel to formula feeding in treating moderate neonatal hypoglycemia. However, the impact of administering 40% dextrose gel immediately after birth to reduce the risk of hypoglycemia during the first two hours of life, while promoting maternal bonding and breastfeeding success at discharge, has not yet been fully evaluated. This study aims to assess the effectiveness of 40% oral dextrose gel in preventing hypoglycemia in at- risk infants during the first 2–4 hours of life. In addition, the study investigates whether this intervention can be employed as a treatment for asymptomatic moderate hypoglycemia (capillary bood glucose level between 25 and 40 mg/dl) occurring within the first two hours of life. Furthermore, the study seeks to determine whether early administration of 40% oral dextrose gel may positively influence breastfeeding outcomes.

## Materials and methods

This was a prospective, randomized, double-arm, single-center, pilot study conducted at the Neonatology Unit of the San Martino Hospital in Genoa, spanning in two phases.

### First phase

The first phase of data collection occurred between November 2020 and January 2022. In this first phase, 200 newborns at risk of hypoglycemia within the first few hours after birth were recruited, either after spontaneous delivery or cesarean section. The newborns were randomly assigned into two parallel groups in a 1:1 ratio, Dextrose group and Control group.

Newborns who met one of the following risk criteria were included: late preterm (LP, gestational age between 34 ^+^ ^0^–36 ^+^ ^6^), LGA, SGA, or those born to mothers with gestational diabetes. Newborns were excluded if they experienced stressful events at birth (e.g., requiring cardiopulmonary resuscitation) were septic, had cardiac or respiratory pathology, were asphyxiated, had congenital defects, genetic disorders, inborn errors of metabolism, or had symptomatic hypoglycemia requiring immediate treatment at birth.

One hundred newborns (Dextrose group) received a prophylactic oral dose of 40% dextrose gel (Destrogel, Orsana®, Italy, 1 vial contains 2 ml of product or 400 mg/ml) at 15 minutes of life while the other 100 newborns (Control group) were managed without receiving the gel.

In Dextrose group, a volume of 2 ml of the product was administered to newborns with a birth weight greater than 2,500 g, whereas those with a birth weight of 2,500 g or less received 1 ml.

Capillary blood glucose level was assessed at 2 and 4 hours of life in all newborns enrolled, using a glucometer. Glycaemia was further checked in case of detection of moderate asymptomatic hypoglycemia at 2 hours of life. Moderate hypoglycemia was defined as capillary blood glucose levels ranging from 25 to 40 mg/dl (4) and was managed differently between the two study groups. Infants in the Dextrose group received an additional dose of 40% oral dextrose gel, whereas those in the Control group were administered 5–10 ml of infant formula (Formula1 for term newborns, or preterm formula for late preterm or small for gestational age infants with a birth weight below 2,500 g). Following oral treatment—either dextrose gel or formula—blood glucose levels were reassessed after 30–60 minutes. In cases of recurrent hypoglycemia, infants were withdrawn from the study and managed according to the Neonatology Unit's internal protocol, in line with the American Academy of Pediatrics (AAP) guidelines (4). Throughout blood glucose monitoring, skin-to-skin contact was encouraged and early initiation of breastfeeding was actively promoted for all infants.

### Second phase

The second phase of data collection took place from February 2022 to April 2024. In this phase, only late preterm newborns with a gestational age between 34 ^+^ ^0^–36 ^+^ ^6^ were randomly assigned into two parallel groups in a 1:1 ratio, Dextrose group and Control group.

Newborns were excluded from the study if they experienced stressful events at birth (such as requiring cardiopulmonary resuscitation), or if they were septic, had cardiac or respiratory pathology, were asphyxiated, had congenital defects, genetic disorders, or inborn errors of metabolism.

A total of 100 LP newborns were included, 50 received 40% dextrose gel (Destrogel, Orsana®, Italy) at 15 minutes after birth (Dextrose group), while 47 patients did not receive the gel (Control group); in the latter group, three newborns were withdrawn from the study within the first hour of life due to the onset of clinical symptoms that warranted exclusion.

As in the first phase of the study, infants randomized to the Dextrose group received 2 ml of the product if their birth weight exceeded 2,500 g, and 1 ml if their birth weight was 2,500 g or less.

Capillary blood glucose was monitored at 2 and 4 hours of life in both groups with glucometer. In case of detection of moderate hypoglycemia at 2 hours of life, patient of both groups received the same treatment according to our Neonatology Unit protocol: a dose of 40% dextrose gel followed by a preterm formula milk meal (5–10 ml), and glucose levels were checked at 30–60 minutes after the correction. If hypoglycemia persisted after oral correction, treatment was provided according to our Department's protocol, and the newborn was removed from the study.

### Dextrose gel administration

Oral dextrose gel was administered by trained staff during skin-to-skin contact to promote maternal bonding without separating the infant from the mother. The gel was applied with the finger massaging the product directly to the oral mucosa, allowing for rapid absorption into the systemic circulation through the lingual and internal jugular veins. Although some of the gel may also be swallowed and absorbed through the gastrointestinal tract, the primary route of absorption is through the mucosal surfaces.

### Data collection

For both phases of the study, a complete database was created containing key characteristics for each newborn: gestational age, birth weight, maternal diabetes status, blood glucose levels at 2 and 4 hours of life, and at 30 and 60 minutes after dextrose gel and/or milk meal administration. Additional data collected included breastfeeding in the first two hours of life, the number of breastfeeding sessions on the first day, feeding status at discharge (exclusive breastfeeding or not), and the need for intravenous glucose solution administration.

This study was approved by the ethical committee on September 30, 2020. Written informed consent was obtained from the parents of all infants prior to any study-related activities. The study was conducted in compliance with the Declaration of Helsinki and in accordance with the Good Clinical Practice.

## Statistical methods

### Sample size

This is a pilot study. At the time of writing the protocol we didn't have literature data about glycaemia at 2 and/or 4 hours after early administration of dextrose gel at 15 minutes of life. We evaluated that a sample of 200 newborns (100 randomly assigned to Dextrose group and 100 to Control group) could be adeguate. In the second phase of the study, based on our previous first phase data and assuming power of 80% and alpha = 0.05, we considered 100 late preterm (50 in each arm, Dextrose or Control group) to be adequate to detect differences in glycaemia at 2 and 4 hours.

### Randomization

New-born who met the inclusion criteria were randomized into the two study arms using a computer-generated Randomization list. Infants were assigned immediately after birth to receive a prophylactic oral dose of 40% dextrose gel (Destrogel, Orsana®, Italy, 1 vial contains 2 ml of product or 400 mg/ml) at 15 minutes of life or without receiving the gel in a 1:1 ratio (First Phase). The study was not blinded, and the staff performing the study also cared for the infants later on but the biostatistician remained blind to the allocation of the study groups. The group assignment was contained in sequentially numbered, sealed, opaque envelopes that were prepared by an independent statistician.

In the second phase of the study, were enrolled only late preterm newborns with a gestational age between 34 + 0–36 + 6, they were randomly assigned into two parallel groups in a 1:1 ratio using a computer-generated Randomization list. Infants were assigned immediately after birth to receive a prophylactic oral dose of 40% dextrose gel (Destrogel, Orsana®, Italy, 1 vial contains 2 ml of product or 400 mg/ml) at 15 minutes of life or without receiving the gel in a 1:1 ratio.

### Statistical analysis

Data were expressed as means and standard deviations (SD) or medians and ranges for continuous variables, and as absolute and relative frequencies for categorical variables. Non-parametric tests (Mann–Whitney *U*-test) were used for continuous variables, and the Chi square or Fisher's exact test was employed for categorical variables to assess differences between groups. Data were stratified by risk categories (LP, SGA, LGA and children of diabetic mother) where necessary. A *p* values ≤ 0.05 was considered statistically significant, with all tests performed as two tailed. Statistical analysis carried out using SPSS for Windows (SPSS Inc, Chicago, Illinois USA).

## Results

### First phase

A total of 200 newborns were enrolled in the first phase of the study. Of these, 100 newborns were assigned to the Dextrose group, and 100 newborns were assigned to the Control group. The characteristics of the recruited population are detailed in Table 1. At a 2-hour glucose check, we observed 16 cases (8%) of moderate hypoglycemia. Among these, 12 cases (12%) occurred in the Dextrose group, while 4 cases (4%) were in the Control group, the difference was not statistically significative (*p* = 0,07). No cases of symptomatic hypoglycemia were noted. Both groups showed an increase in blood glucose levels during the follow-up measurement conducted 30–60 minutes after oral treatment: the Dextrose group received a second dose of dextrose, while the Control group was given an oral milk meal. Among the newborns with moderate hypoglycemia, 8 out of 16 (50%) required intravenous glucose infusion and were subsequently excluded from the study.

**Table 1:** Descriptive characteristics of the population of the first phase of the study.

Population characteristics	All (N = 200)	Dextrose (N = 100)	Control (N = 100)
	Mea*n* ± SD	Mean ± SD	Mean ± SD
Gestational age, *weeks*	38.86 ± 3.19	39.07 ± 1.51	38.66 ± 4.25
Birth weight, *g*	3,213.6 ± 696.4	3,253.3 ± 693.3	3,173.8 ± 700.6
Weight, *centile*	47.22 ± 39.09	50.13 ± 39.39	44.30 ± 38.8
	*N* (%)	*N* (%)	*N* (%)
Gender, *male*	110 (55)	55 (50)	55 (50)
Gender, *female*	90 (45)	45 (50)	45 (50)
SGA	64 (32)	31 (48.4)	33 (51.6)
LGA	63 (31.5)	31 (49.2)	32 (50.8)
DMG	70 (35)	40 (57.1)	30 (42.9)
GA <37 weeks	29 (14.5)	12 (41.4)	17 (58.6)

**Figure 1:**
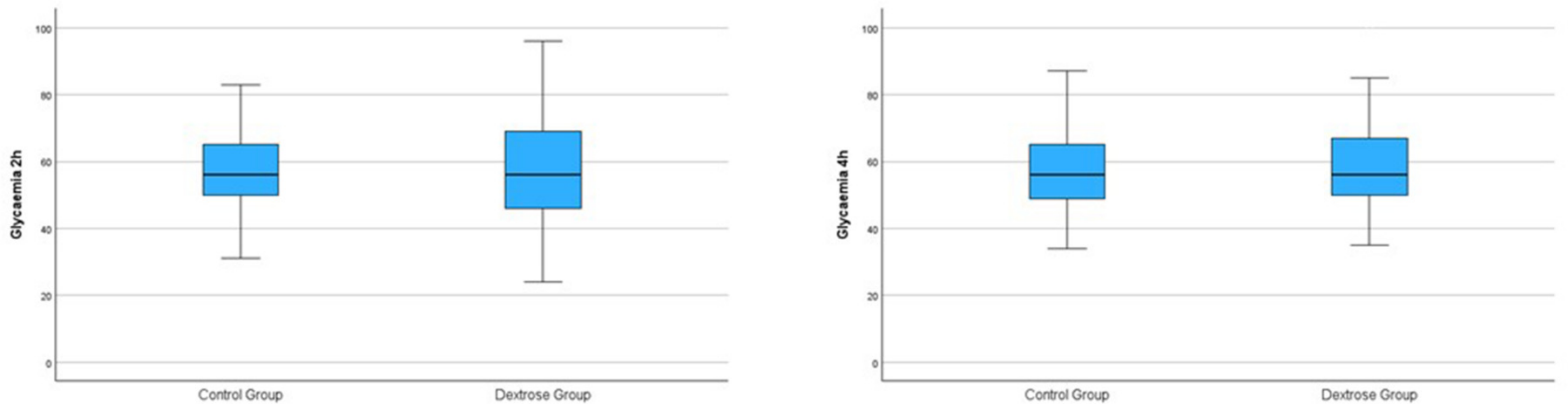
Blood glucose values at 2 and 4 hours for both the dextrose and control groups.

At the 2-hour glucose check, no significant difference in blood glucose levels was found between the Dextrose and Control groups (57.23 ± 15.93 vs. 57.72 ± 12.59; *p* = 0.78) (Table 2 and Figure 1). At the 4-hour glucose check, blood glucose levels in the Dextrose group tended to increase more compared to the Control group, although the difference was not statistically significant (Table 2 and Figure 1). There were no significant differences between the two groups in terms of breastfeeding within the first two hours of life or the number of breastfeeding episodes during the first 24 hours of life (Table 2).

**Table 2:** First phase: glycaemia values and breastfeeding data in the total population comparing dextrose group and control group.

Outcome measures	All (*N* = 200)	Dextrose (*N* = 100)	Control (*N* = 100)	*P*-value
	Mean ± SD	Mean ± SD	Mean ± SD	
Glycaemia 2 h	57.48 ± 14.32	57.23 ± 15.93	57.72 ± 12.59	0.78
Glycaemia 4 h	58.29 ± 12.24	59.15 ± 12.91	57.42 ± 11.53	0.42
	*N* (%)	*N* (%)	*N* (%)	*P*-value
Breastfeeding within 2nd hour	117 (58.5)	56 (47.9)	61 (52.1)	0.57
	Mean ± SD	Mean ± SD	Mean ± SD	*P*-value
*N* of breastfeeding attempts within 24 h	4.97 ± 3.16	5.04 ± 3.15	4.91 ± 3.18	0.80

Blood glucose levels at 2 and 4 hours of life were further analyzed within different at-risk subgroups: SGA, LGA, infants of diabetic mothers, and late preterm infants.

For SGA newborns, no significant differences in blood glucose levels at 2 hours (56.94 ± 14.82 vs. 59.06 ± 13.55, *p* = 0.59) or at 4 hours (57.32 ± 11.40 vs. 53.18 ± 10.07, *p* = 0.13) were observed between the Dextrose and Control groups. However, blood glucose levels in the Control group significantly decreased between the two time points (from 59.06 ± 13.55 to 53.18 ± 10.07, *p* = 0.04), whereas in the Dextrose group, levels tended to increase slightly, but not significantly (from 56.94 ± 14.82 to 57.32 ± 11.40, *p* = 0.77) (Table 3).

**Table 3:** First phase: comparison of glycaemia values between 2 and 4 h in dextrose group and control group in each at-risk subgroups.

Risk subgroup	Dextrose				Control			
		Glycaemia 2 h	Glycaemia 4 h			Glycaemia 2 h	Glycaemia 4 h	
	*N*	Mean ± SD	Mean ± SD	*P*-value	*N*	Mean ± SD	Mean ± SD	*P*-value
SGA	31	56.94 ± 14.82	57.32 ± 11. 04	0.77	33	59.06 ± 13.55	53.18 ± 10.07	**0.04**
LGA	31	56.71 ± 14.53	58.42 ± 11.76	0.47	32	59.97 ± 11.36	57.94 ± 9.11	0.63
DMG	40	57.33 ± 15.62	60.83 ± 14.13	0.30	30	54.97 ± 13.13	61.72 ± 12.86	**0.02**
GA < 37 weeks	12	43.00 ± 17.45	60.08 ± 15.97	**0.008**	17	52.06 ± 13.25	61.82 ± 8.60	**0.01**

Bold values indicate *P*-value ≤ 0.05 is considered statistically significant.

For LGA newborns and infants of diabetic mothers, no significant differences in blood glucose levels were found between the two groups at either 2 or 4 hours of life.

For late preterm, blood glucose levels at 2 hours, were lower in the Dextrose group compared to the Control group (43.00 ± 17.45 vs. 52.06 ± 13.25, *p* = 0.07), but this difference was not statistically significant. Both groups showed a significant increase in blood glucose levels between the two time points (Dextrose: from 43.00 ± 17.45 to 60.08 ± 15.97, *p* = 0.008; Control: from 52.06 ± 13.25 to 61.82 ± 8.60, *p* = 0.01), with the increase being more pronounced in the Dextrose group (Table 3).

### Second phase

In the second phase of the study, 100 late preterm newborns were enrolled: 50 newborns were assigned to the Dextrose group and 50 to the Control group, in the latter group, three newborns were withdrawn from the study within the first hour of life due to the onset of clinical symptoms that warranted exclusion.

The characteristics of the enrolled patients are detailed in Table 4. Among these patients, some had additional risk factors, such as being SGA (11%), LGA (7%), or being born to diabetic mothers (19%), in addition to prematurity (Table 4).

**Table 4:** Descriptive characteristics of the population of the second phase of the study.

Population characteristics	Total (*N* = 97)		Dextrose (*N* = 50)		Control (*N* = 47)	
	Mean ± SD	Min-max	Mean ± SD	Min-max	Mean ± SD	Min-max
Gestational age, weeks	36 ± 0.8	34–36.6	36 ± 0.7	34–36.9	36 ± 0.8	34–36.9
Birth weight, g	2,507 ± 392	1,500–3,670	2,493 ± 398	1,915–3,670	2,523 ± 389	1,500–3,270
Weight, centile	41.5 ± 29	2–99	39.4 ± 29.1	3–99	43.7 ± 38.8	2–98
	*N* (%)		*N* (%)		*N* (%)	
SGA	11 (11)	–	5 (45)	–	6 (55)	–
LGA	7 (7)	–	4 (57)	–	3 (43)	–
DMG	19 (19)	–	10 (53)	–	9 (47)	–

No cases of severe hypoglycemia or symptomatic hypoglycemia requiring immediate intravenous glucose infusion were observed. Eighteen cases (18.6%) of moderate asymptomatic hypoglycemia were noted at 2 hours of life, with 9 cases (18%) in the Dextrose group and 9 cases (19.1%) in the Control group. In both groups, blood glucose levels significantly increased 30–60 minutes following oral administration of dextrose gel and a preterm formula meal (from 32.22 ± 5.67 to 43.44 ± 12.36, *p* = 0.01).

Among the 18 newborns with moderate hypoglycemia at 2 hours of life, 61% (11/18) required intravenous glucose infusion after oral treatment, with 6 infants in the Dextrose group and 5 in the Control group requiring infusion. At the 2- hour blood glucose check, the Dextrose group had higher mean blood glucose levels compared to the Control group, although this difference was not statistically significant (57.62 ± 17.97 vs. 52.94 ± 15.76; *p* = 0.10). Similarly, at the 4-hour glucose check, higher blood glucose levels were observed in the Dextrose group, but the difference again was not statistically significant (69.64 ± 21.11 vs. 65.30 ± 16.18; *p* = 0.42) (Table 5). When excluding infants who required intravenous glucose infusion at any point during the 4- hour monitoring period, the Dextrose group had significantly higher blood glucose levels at 2 hours compared to the Control group (58.48 ± 17.33 vs. 51.28 ± 12.08; *p* = 0.03). For the 4-hour check, a trend toward higher blood glucose levels in the Dextrose group was noted, although the difference was not statistically significant (66.28 ± 17.8 vs. 60.75 ± 13.42; *p* = 0.19) (Table 5).

**Table 5:** Second phase: comparison of glycaemia values at 2 and 4 h in dextrose group and control group in all LP and in LP who did not receive a glucose infusion.

Glycaemic measurement	Total (*N* = 97)		Dextrose (*N* = 50)		Control (*N* = 47)		
	Mean ± SD	min–max	Mean ± SD	min–max	Mean ± SD	min–max	*P*-value
Glycaemia 2 h (mg/dl)	55.35 ± 17.01	24–109	57.62 ± 17.97	24–103	52.94 ± 15.76	25–109	0.10
Glycaemia 4 h (mg/dl)	67.54 ± 19.17	30–137	69.64 ± 21.11	30–137	65.30 ± 16.18	37–111	0.42
	Total without infusion (*N* = 76)		Dextrose without infusion (*N* = 40)		Control without infusion (*N* = 36)		
	Mean ± SD	min–max	Mean ± SD	min–max	Mean ± SD	min–max	*P*-value
Glycaemia 2 h (mg/dl)	55.07 ± 15.41	24–103	58.48 ± 17.33	24–103	51.28 ± 12.08	31–85	0.03
Glycaemia 4 h (mg/dl)	63.66 ± 16.01	30–111	66.28 ± 17.8	30–111	60.75 ± 13.42	37–95	0.19

Between the second and fourth hour, blood glucose levels tended to be higher on average in the Dextrose group (Dextrose group: 57.62 ± 17.97 vs. 69.64 ± 21.11, *p* ≤ 0.001; Control group 52.94 ± 15.76 vs. 65.30 ± 16.80, *p* ≤ 0.001). In patients who did not receive an infusion, an increase in blood glucose levels was observed between the second and fourth hour in both groups; however, this increase reached statistical significance only in the control group (Table 6).

**Table 6:** Second phase: Glycaemia values between 2 and 4 h in Dextrose group and Control group in all LP and LP who did not receive a glucose infusion.

Subgroup	Dextrose			Control				
		Glycaemia 2 h (mg/dl)	Glycaemia 4 h (mg/dl)			Glycaemia 2 h (mg/dl)	Glycaemia 4 h (mg/dl)	
	*N*	Mean ± SD	Mean ± SD	*P*-value	*N*	Mean ± SD	Mean ± SD	*P*-value
Total LP	50	57.62 ± 17.97	69.64 ± 21.11	≤0.001	47	52.94 ± 15.76	65.30 ± 16.80	≤0.001
LP without infusion	40	58.48 ± 17.33	66.28 ± 17.8	0.07	36	51.28 ± 12.08	60.75 ± 13.42	0.003

No differences were observed between the two groups regarding breastfeeding within the first two hours of life or the number of breastfeeding sessions within the first 24 hours. However, among infants who had skin-to-skin contact postpartum and latched within the first two hours, the Dextrose group had significantly higher blood glucose levels at 2 hours compared to the Control group (66.33 ± 15.86 vs. 55.07 ± 14.32, *p* = 0.05). This difference was not observed in those who did not latch within the first two hours of life (Table 7). No side effects were observed from the use of dextrose gel in either phase of the study.

**Table 7:** Second phase: blood glucose at 2 h post breastfeeding or not breastfeeding within the first two hours of life in dextrose group and control group.

Glycaemia post breastfeeding or not breastfeeding	Total (*N* = 97)		Dextrose (*N* = 50)		Control (*N* = 47)		
	*N*	Mean ± SD	*N*	Mean ± SD	*N*	Mean ± SD	*P*-value
2-hour blood glucose preceded by breastfeeding	26	60.27 ± 15.82	12	66.33 ± 15.86	14	55.07 ± 14.32	0.05
2-hour blood glucose NOT preceded by breastfeeding	71	53.55 ± 17.18	38	54.87 ± 17.9	33	52.03 ± 16.46	0.40

## Discussion

A prompt diagnosis and treatment of hypoglycemia are essential for newborn management, as the long-term effects of this disorder remain uncleared. Given the neurological risks associated with hypoglycemia, immediate intervention is crucial. In this context, the use of oral dextrose gel has become an important therapeutic option for preventing and treating hypoglycemia in at-risk newborns. Previous studies, such as the 2013 study (18) demonstrated that a dose of 200 mg/kg of 40% dextrose gel, administered with either breastfeeding or formula milk was effective in treating asymptomatic hypoglycemia in newborns with major risk factors (infants of diabetic mothers, SGA, LGA, and mild prematurity with gestational age between 35 + 0 and 36 + 6 weeks). This approach was shown to maintain adequate glucose levels and reduce the need for NICU admissions and intravenous glucose infusions. A positive effect on the initiation of breastfeeding was also observed. These results have been confirmed by subsequent studies (18, 19, 24, 28).

Recent studies have focused on the effectiveness of early and prophylactic dextrose gel administration (22, 25–27). Specifically, Hegarty et al. (26) demonstrated that different doses of 40% dextrose gel (0.5 ml/kg = 200 mg/kg or 1 ml/kg = 400 mg/kg), administered early as prophylaxis in at-risk newborns—infants of diabetic mothers, SGA, LGA, and late preterm—at one hour of life, once or followed by three additional doses of 0.5 ml/kg = 200 mg/kg within the first 12 hours of life) were effective in reducing the risk of hypoglycemia. Similarly, Harding et al. (25) found that a single dose of 0,5 m/kg 40% dextrose gel at one hour of life was effective in preventing hypoglycemia, although it did not significantly reduce NICU admissions. Moreover, Griffith et al. (29) conducted a two-year follow-up study in 2021, confirming that prophylactic dextrose gel did not cause any long-term side effects, including no clinically relevant impacts on neuro-motor or sensory outcomes.

In our study, we explored the effect of early dextrose gel administration in at-risk newborns without necessarily combining it with a meal. While we confirmed previous findings in certain groups (e.g., SGA and late preterm newborns), we found that dextrose gel administration did not have a significant impact on glucose levels in LGA infants and those of diabetic mothers.

A preliminary analysis of 200 at-risk newborns (LGA, SGA, infants of diabetic mothers, late preterm) revealed different responses to dextrose gel compared to previous studies by Harding (25) and Hegarty (26). In LGA infants and those of diabetic mothers, dextrose gel did not significantly affect glucose levels in the first few hours of life. However, in late preterm and SGA infants dextrose gel had a positive impact on glucose levels in the hours following birth, aligning with Harding et al.'s findings. Specifically, glucose levels in these newborns improved between the second and fourth hours of life, indicating a role for dextrose gel in maintaining stable glucose levels in the early hours.

We believe this difference in response is due to varying mechanisms of hypoglycemia in these populations. In infants of diabetic mothers and LGA newborns, hyperinsulinism is the primary cause, whereas in SGA and preterm infants, energy reserve depletion and immature compensatory mechanisms are more likely at play. Based on these preliminary results, which suggested higher glucose levels over time in late preterm and SGA infants, we decided to expand the sample by recruiting more late preterm newborns, a group underrepresented in the initial phase of the study.

Analysis of the expanded sample showed that dextrose gel administration resulted in significantly higher glucose levels at two hours in late preterm newborns, consistent with previous studies (25, 26). Notably, none of the infants who received the gel experienced side effects, including rebound hypoglycemia. The newborns tolerated the gel well, with no increase in regurgitation or other signs of intolerance. Importantly, dextrose gel did not interfere with breastfeeding. In fact, newborns who were breastfed within the first two hours of life had significantly higher glucose levels. However, there was no significant increase in the number of breastfeeding sessions within the first 24 hours, suggesting the gel does not directly stimulate suckling. This finding implies that dextrose gel could help maintain safe glucose levels without affecting breastfeeding and, as a simple, cost-effective intervention with no side effects, it could be beneficial during skin-to-skin contact.

The main limitation of this study is the relatively small sample size and the low statistical power for some outcomes. When we designed the study, there was no literature available regarding the administration of the glucose gel at 15 minutes of life, so it was not possible to determine an adequate sample size.

Our study is a pilot study, in the first phase the statistical power was found to be low (<80%) regarding the blood glucose variable at 2 hours, while for blood glucose at 4 hours was 95%. In the second phase, however, power regarding the blood glucose variable at 2 hours and blood glucose at 4 hours was 95%.

Furthermore, the study population was characterized by a certain degree of heterogeneity, as several participants presented with multiple concurrent risk factors for neonatal hypoglycemia (e.g., prematurity, maternal diabetes, SGA, LGA). This heterogeneity, particularly evident within the late preterm subgroup may have influenced the outcomes and posed challenges in isolating the specific effect of 40% oral dextrose gel. The subsequent expansion of the late preterm cohort in the second phase of the study partially mitigated this limitation.

Despite these constraints, the study population was carefully selected to minimize the presence of confounding variables.

Another limitation is the absence of data on blood glucose levels and hypoglycemia beyond 4 hours of life thus no statement can be made about the prevention of hypoglycemia occurring at a later stage.

Our study did not include data on the long-term effects of dextrose gel on growth and neurodevelopmental outcomes, making further monitoring essential.

In conclusion, our findings suggest that dextrose gel, in addition to its established role in treating moderate asymptomatic hypoglycemia when combined with a meal, could be effective in preventing hypoglycemia and maintaining adequate glucose levels in the first hours of life. This is particularly relevant for SGA and late preterm newborns when administered early after birth and followed by breastfeeding. In SGA infants, a trend toward higher glucose levels at four hours of life was observed, while in late preterm newborns, the positive impact seems to occur earlier. Further studies, preferably with larger sample sizes, are needed to confirm these results and better understand the long-term effects of dextrose gel.

## Data Availability

The original contributions presented in the study are included in the article/Supplementary Material, further inquiries can be directed to the corresponding author.

## References

[1] Adamkin DH. Neonatal hypoglycemia. Semin Fetal Neonatal Med. (2017) 22(1):36–41. 10.1016/j.siny.2016.08.007 27605513

[2] Harding JE Alsweiler JM Edwards TE McKinlay CJ. Neonatal hypoglycaemia. BMJ Med. (2024) 3(1):e000544. 10.1136/bmjmed-2023-000544 38618170 PMC11015200

[3] Harris DL Weston PJ Gamble GD Harding JE. Glucose profiles in healthy term infants in the first 5 days: the glucose in well babies (GLOW) study. J Pediatr. (2020) 223:34–41.e4. 10.1016/j.jpeds.2020.02.079 32381469

[4] Adamkin DH, Committee on Fetus and Newborn. Postnatal glucose homeostasis in LatePreterm and term infants. Pediatrics. (2011) 127(3):e20103851. 10.1542/peds.2010-3851 21357346

[5] Bertino E Spada E Occhi L Coscia A Giuliani F Gagliardi L Neonatal anthropometric charts: the Italian neonatal study compared with other European studies. J Pediatr Gastroenterol Nutr. (2010) 51(3):353–61. 10.1097/MPG.0b013e3181da213e 20601901

[6] Alsaleem M Saadeh L Kamat D. Neonatal hypoglycemia: a review. Clin Pediatr (Phila). (2019) 58(13):1381–6. 10.1177/0009922819875540 31556318

[7] Stanley CA Rozance PJ Thornton PS Leon D Harris DD Haymond D Reevaluating “transitional neonatal hypoglycemia”: mechanism and implications for management. J Pediatr. (2015) 166(6):1520–1525.e1. 10.1016/j.jpeds.2015.02.045 25819173 PMC4659381

[8] Thompson-Branch A Havranek T. Neonatal hypoglycemia. Pediatr Rev. (2017) 38(4):147–57. 10.1542/pir.2016-0063 28364046

[9] Cornblath M Hawdon JM Williams AF Aynsley-Green A Ward-Platt MP Schwartz R Controversies regarding definition of neonatal hypoglycemia: suggested operational thresholds. Pediatrics. (2000) 105(5):1141–5. 10.1542/peds.105.5.1141 10790476

[10] De Angelis LC Brigati G Polleri G Malova M Parodi A Minghetti D Neonatal hypoglycemia and brain vulnerability. Front Endocrinol. (2021) 12:634305. 10.3389/fendo.2021.634305 PMC8008815 33796072

[11] Gu MH Amanda F Yuan TM. Brain injury in neonatal hypoglycemia: a hospital-based cohort study. Clin Med Insights Pediatr. (2019) 13:1179556519867953. 10.1177/1179556519867953 31447599 PMC6688136

[12] Tam EWY Haeusslein LA Bonifacio SL Glass HC Rogers EE Jeremy RJ Hypoglycemia is associated with increased risk for brain injury and adverse neurodevelopmental outcome in neonates at risk for encephalopathy. J Pediatr. (2012) 161(1):88–93. 10.1016/j.jpeds.2011.12.047 22306045 PMC3346850

[13] Thornton PS. Neonates at risk for hypoglycemia: associated neurological outcomes. J Pediatr. (2016) 170:341–4. 10.1016/j.jpeds.2015.12.057 26922771

[14] Levene I Wilkinson D. Identification and management of neonatal hypoglycaemia in the fullterm infant (British association of perinatal medicine—framework for practice). Arch Dis Child Educ Pract Ed. (2019) 104(1):29–32. 10.1136/archdischild-2017-314050 29903743

[15] Queensland Clinical Guidelines. Hypoglycaemia–newborn. Queensland: Queensland Clinical Guidelines Steering Committee Statewide Maternity and Neonatal Clinical Network (2023). Available at: https://www.health.qld.gov.au/__data/assets/pdf_file/0043/881899/g-hypogly.pdf

[16] Wackernagel D Gustafsson A Edstedt Bonamy AK Reims A Ahlsson F Elfving M Swedish National guideline for prevention and treatment of neonatal hypoglycaemia in newborn infants with gestational age ≥35 weeks. Acta Paediatr. (2020) 109(1):31–44. 10.1111/apa.14955 31350926

[17] Wight NE, Academy of Breastfeeding Medicine. ABM Clinical protocol #1: guidelines for glucose monitoring and treatment of hypoglycemia in term and late preterm neonates, revised 2021. Breastfeed Med. (2021) 16(5):353–65. 10.1089/bfm.2021.29178.new 33835840

[18] Harris DL Weston PJ Signal M Chase JG Harding JE. Dextrose gel for neonatal hypoglycaemia (the sugar babies study): a randomised, double-blind, placebo-controlled trial. Lancet. (2013) 382(9910):2077–83. 10.1016/S0140-6736(13)61645-1 24075361

[19] Meneghin F Manzalini M Acunzo M Daniele I Bastrenta P Castoldi F Management of asymptomatic hypoglycemia with 40% oral dextrose gel in near term at-risk infants to reduce intensive care need and promote breastfeeding. Ital J Pediatr. (2021) 47(1):201. 10.1186/s13052-021-01149-7 34627324 PMC8500822

[20] Rawat M Chandrasekharan P Turkovich S Barclay N Perry K Schroeder E Oral dextrose gel reduces the need for intravenous dextrose therapy in neonatal hypoglycemia. Biomed Hub. (2016) 1(3):1–9. 10.1159/000448511 27840813 PMC5104273

[21] Harris DL Gamble GD Weston PJ Harding JE. What happens to blood glucose concentrations after oral treatment for neonatal hypoglycemia? J Pediatr. (2017) 190:136–41. 10.1016/j.jpeds.2017.06.034 28709629

[22] Devarapalli V Niven M Canonigo J Spray B Avulakunta I Beavers J Prophylactic dextrose gel use in newborns at risk for hypoglycemia. J Perinatol. (2024) 44(11):1640–6. 10.1038/s41372-024-02133-9 39363038

[23] Lamy E Orneto C Ali OHA Kireche L Mathias F Bouguergour C Formulation, quality control and stability study of pediatric oral dextrose gel. Pharmaceuticals (Basel). (2025) 18(2):204. 10.3390/ph18020204 40006018 PMC11858957

[24] Edwards T Liu G Battin M Harris DL Hegarty JE Weston PJ Oral dextrose gel for the treatment of hypoglycaemia in newborn infants. Cochrane Database Syst Rev. (2022) 3(3):CD011027. 10.1002/14651858.CD011027.pub3 35302645 PMC8932405

[25] Harding JE Hegarty JE Crowther CA Edlin RP Gamble GD Alsweiler JM Evaluation of oral dextrose gel for prevention of neonatal hypoglycemia (hPOD): a multicenter, doubleblind randomized controlled trial. PLoS Med. (2021) 18(1):e1003411. 10.1371/journal.pmed.1003411 33507929 PMC7842885

[26] Hegarty JE Harding JE Gamble GD Crowther CA Edlin R Alsweiler JM. Prophylactic oral dextrose gel for newborn babies at risk of neonatal hypoglycaemia: a randomised controlled dose-finding trial (the Pre-hPOD study). PLoS Med. (2016) 13(10):e1002155. 10.1371/journal.pmed.1002155 27780197 PMC5079625

[27] Roberts L Lin L Alsweiler J Edwards T Liu G Harding JE. Oral dextrose gel to prevent hypoglycaemia in at-risk neonates. Cochrane Database Syst Rev. (2023) 11(11):CD012152. 10.1002/14651858.CD012152.pub4 38014716 PMC10683021

[28] Gibson BL Carter BM LeDuff LD Wallace A. 40% glucose gel for the treatment of asymptomatic neonatal hypoglycemia. Adv Neonatal Care. (2021) 21(5):371–8. 10.1097/ANC.0000000000000823 33350707

[29] Griffith R Hegarty JE Alsweiler JM Gamble GD May R McKinlay CJD Two-year outcomes after dextrose gel prophylaxis for neonatal hypoglycaemia. Arch Dis Child Fetal Neonatal Ed. (2021) 106(3):278–85. 10.1136/archdischild-2020-320305 33148686 PMC8062278

